# Perceived barriers to methadone maintenance treatment among Iranian opioid users

**DOI:** 10.1186/s12939-018-0787-z

**Published:** 2018-06-11

**Authors:** Maryam Khazaee-Pool, Maryam Moeeni, Koen Ponnet, Arezoo Fallahi, Leila Jahangiri, Tahereh Pashaei

**Affiliations:** 1 Department of Health Education and Promotion, School of Public Health, Zanjan University of Medical Sciences, Zanjan, Iran; 2 Health Management and Economics Research Center, Isfahan University of Medical Sciences, Isfahan, Iran; 3 Department of Communication Sciences, imec-mict-Ghent University, Ghent, Belgium; 4 Enviromental Health Research Center, Research Institute for Health Development, Kurdistan University of Medical Science, Sanandaj, Iran; 5 Research Center for Evidence Based Medicine, Tabriz University of Medical Sciences, Tabriz, Iran; 6 Department of Public Health, Faculty of Health, Kurdistan University of Medical Sciences, Sanandaj, Iran

**Keywords:** Perceived barriers, Methadone maintenance treatment, Opioid users

## Abstract

**Background:**

Opioid use is a severe problem in Iran. Despite methadone maintenance treatment (MMT) programs being one of the most important treatment strategies for reducing individual and public harms associated with opioid use, a large proportion of Iranian patients refuse to participate in such treatment programs.

**Methods:**

The present study aims to explore the beliefs and attitudes toward MMT programs of opioid-dependent patients who were participating or had participated in methadone therapy. In-depth interviews were conducted with 23 opioid users between 27 and 58 years of age from Kurdistan provinces.

**Results:**

Overall, six themes were discovered to be key barriers relating to methadone treatment, including financial barriers related to methadone treatment, lack of awareness about methadone treatment, negative attitudes regarding using methadone, worries about methadone’s side effects, social stigma ascribed to methadone therapy, and systemic barriers to methadone treatment.

**Conclusion:**

Our study revealed that the cost of treatment is a major obstacle to attending and continuing at MMT programs and that addicts and their families are not always accurately informed about the duration of MMT programs and the side effects of methadone treatment.

## Background

The United Nations Office on Drugs and Crime (UNODC) reported that in 2013, the universal prevalence of opioid use among adults was around 0.7%, which ranked opioids as the second most common form of illicit drug used worldwide. Opioid use has a long history in Iran, as a result of which there is some level of social tolerance toward it in some regions [1, 2]. Although opium is the dominant type of opioid used in Iran, over time, the pattern of opioid use in Iran has changed into other types of opioids, such as shireh, heroin, and kerack-heroin [1]. Opioid use harms individuals physically and psychiatrically, imposes economic and social burdens on society and raises huge public health concerns in Iran [3] as well as internationally [4–12]. Beyond the health burdens of opioid use disorder on individuals and communities, dependence on drugs hinders economic productivity and diminishes human capital [8, 13]. In addition, opioid markets increase illegal careers and negatively affect the resources available in legal economies [8, 12]. Illegal opioid markets are also linked to rises in crime and social insecurity [5].

Methadone maintenance therapy (MMT) is the most frequently used opioid therapy. It is readily available for patients seeking opioid treatment in many countries [14–16], and it was implemented in 60 out of 70 countries providing opioid treatment services (OTS) in 2009 [11]. Stable, prolonged MMT yields prominent benefits to both patients and communities [15, 17]. Scientific evidence has demonstrated that MMT has the capacity to reduce the need to use opioids, particularly opioid injection [6, 9, 16, 18]; decrease the adverse health effects of drug use, such as fatal and nonfatal drug overdoses [12, 19, 20]; and keep patients in treatment and decrease the risk of relapse to drug use [12, 18]. Furthermore, MMT seems to improve HIV treatment outcomes [6, 7, 9], reduce HIV and hepatitis transmission [6, 13, 19], control dysfunctional behaviors [12], and suppress criminal activities, notably drug-related crimes such as drug dealing [6, 13, 21]. Studies have also shown that stabilizing patients in MMT programs provides them with a better chance to find and hold a suitable job [16], work more productively, build strong family and social relations [16, 22], and enhance their quality of life [16, 22, 23]. All these returns ultimately have a positive influence on public health and security as well as human capital and social productivity [5, 8, 16]. MMT is also a more cost-effective treatment intervention than other opioid maintenance treatments. For instance, a research study conducted in California during 2000 and 2001 found that substance abuse treatment has a benefit-to-cost ratio greater than 7:1 [24]. There is evidence that in low- and middle-income countries where there is a lack of treatment programs, the expansion of MMT programs might lead to savings in social and health expenditures [12, 19, 25].

In recent years, more than 80% of the recognized drug-treatment seekers in Iran were primarily dependent on opioids [3, 26]. Although buprenorphine maintenance therapy (BMT), a well-known type of OST, is now available in Iran, MMT programs are the most frequently used therapy [1, 11]. Originally, MMT programs were launched in Iran as a harm-reduction initiative. After a successful pilot test in 2002 in the Iranian National Center for Addiction Studies (INCAS), the MMT program has implemented in public and private clinical centers. By the end of 2009 there were approximately 16,000 clinical sites established by the Ministry of Health nationwide that provided MMT program services for 159,000 opioid-dependent patients according to treatment protocols [1]. The cost of MMT services is different in governmental and private centers. On average, patients have to pay $20–$30 monthly in governmental centers. This fee is considerably higher in private centers.

Even though MMT programs are one of the most important treatment strategies for reducing individual and public harms associated with opioid use, and despite the central role of MMT in harm-reduction approaches to opioid use in Iran and many other countries, previous studies have pointed out that a large proportion of eligible patients refuse to participate in this type of treatment program [4, 5, 13, 27, 28]. What is more, many participating patients seem to drop out of such programs [5, 28]. According to evidence, various obstacles, notably the perception of barriers related to MMT as well as misconceptions that opioid replacement treatment can cure the addiction problem in the short run, negatively affect patients’ entrance and adherence to MMT programs, respectively, and hinder satisfying treatment outcomes [4–6, 13, 16, 19, 28]. Better knowledge about the obstacles perceived by opioid users could provide policy makers and practitioners with a guide to attracting a greater proportion of opioid patients to treatment programs and ensuring patients’ compliance with treatment [5]. To the best of our knowledge, no study has focused on the obstacles to entering MMT programs from the perspective of opioid-dependent patients in Iran. To fill this gap in the existing literature, the present qualitative study aims to explore the beliefs and attitudes toward MMT programs of opioid-dependent patients who were participating or had participated in an MMT.

## Methods

### Participants

Between February and July 2016, we conducted 23 in-depth qualitative interviews with opioid users in Kurdistan province, Iran. About half (*n* = 12) of the participants were users who were currently in MMT. Seven of the other participants had formerly been using methadone but dropped out of MMT, and four of the other opioid users had never begun MMT. An Institutional Review Board (IRB) approved this research, and ethical approval was obtained from the Kurdistan University of Medical Sciences IR.94/97. Written informed consent was also obtained from all participants.

The inclusion criteria for this study were: (1) being 20 years old or older and (2) being an opioid user. In order to gain different viewpoints, a purposive sampling method with maximum variation was used. Four MMT clinics were randomly chosen from a total of 10 MMT clinics in the Kurdistan province. To gain maximum variation, we opted to recruit opioid users from different age groups with different socioeconomic, educational, and occupational levels, varying religiosity, and different marital statuses.

#### Data collection

Open-ended questions were used in the 23 in-depth interviews, which were performed using a semi-structured interview guide. Each interview started with an opening question in which the researcher asked the respondents about their experiences with MMT. Depending on the topics that were brought up by the respondents, the researcher chose other questions that touched on their experiences with MMT. Examples of questions were “Do you consider MMT to be efficacious?”, “What problems have you experienced with MMT?”, “If you discontinued the treatment, what were the reasons and problems that you experienced?”, “What positive or negative experiences did you gain from treatment with methadone?”, “Did you experience side effects from methadone, and if so, which side effects?” and “What was the perspective of family and friends on the regular use of methadone as a treatment?” Depending on the participants’ responses, the moderators rephrased some questions or asked additional questions if they wanted to delve deeper into specific issues that were brought up by the respondents. Examples included “What do you mean?” and “Can you explain this more?” We recorded our analytical concept by memo writing. Each interview lasted for approximately 1.5 to 2 h and was conducted in an isolated room by an expert interviewer. Data were collected until saturation was obtained.

#### Data analysis

Data were analyzed with MAXQDA software using Graneheim and Lundman’s method [29]. A qualitative content analysis with a conventional approach was applied to the information gained from the semi-structured interviews in order to detect the semantic units. Immediately after the interviews, the recorded interviews were typewritten. Transcriptions were analyzed and coded so that the next interviews were directed by information obtained from the previous interviews. The following steps were applied for qualitative data analysis. First, the transcripts were read and reread by the researchers, and then important quotes were highlighted. In the following step, meaning units were produced from the statements. In order to elicit primary codes, a comparative analysis was applied. Then, themes and subthemes were constructed based on the codes with similar meanings.

## Results

### Description of study sample

The twenty-three opioid users who participated in the present study were between 27 and 58 years old with a mean age of 43.27 years. Due to a lack of cooperation from treatment centers in which female drug users are treated, this study was conducted among male drug users only. Most of the men (*n* = 15) were married, and less than a quarter (*n* = 3; 13%) had never been married. The participants had various levels of education: five (21.6%) had completed higher education, four (17.4%) had completed secondary or high school education, and 14 (61%) had completed fewer than six years of education (primary school). Fourteen participants (61%) were employed. All but two participants used tobacco, seven used crack-heroin on a regular basis, 12 reported that they used heroin, and four used other illegal drugs.

Overall, six main themes were discovered as key barriers relating to MMT, including financial barriers related to MMT, lack of awareness about MMT, negative attitudes toward using methadone, worries about methadone side effects, social stigma ascribed to MMT, and systemic barriers to MMT.

### Theme #1: Financial barriers related to methadone treatment

Although some participants had accepted entering treatment, they did not enter treatment because of financial problems, and as a result they continued using drugs and were unable to get MMT. Unemployment and the high cost of MMT were the biggest barriers to entering treatment for some participants. Patients had to expend more cash, which came mostly from their families, and it was common for them to request more money from their families, which may have placed a financial burden on their family.

*“I’m speaking about myself… As a known addicted person I couldn’t have a good job, thus any income... I couldn’t pay the methadone fee. My father wants to financially support the treatment cost, but he makes below 7000,000 rial per month. Methadone costs 1,300,000 rial per month, not counting transportation. How much is needed for their day-to-day costs? He is getting older … what do I do after he dies?”* (P7; 58 years old, divorced, high school education, currently using MMT, a heroin user).

The participants without work were also dependent on family members to support their MMT. Some participants who did not get financial support from their family were not able to be treated with methadone. Besides the treatment fee, the day-to-day transportation costs were also a financial burden. As three of the patients reported, *“My wife provides for our living expenses with carpet weaving and crafts, which pay little. The price of the cure is very high, 40,500 rials every day. I have a good family and they are really supportive, but my family’s financial ability is too poor ... and because I’m unemployed, it is really hard for me.”* (P11; 42 years old, married, university education, currently using MMT, a heroin user).

Another man said, *“I had a lot of problems during treatment: unemployment and lack of money because of this, the cost of renting a house. Sometimes I even did not pay for treatment*, *because the cost of MMT was not so much lower and I could pay for drugs instead. The government does not support us for the cost of treatment, but I have a good family. They are really supportive, but my family’s financial ability is poor. And because I’m unemployed ... it is really hard for me.”* (P7; 55 years old, divorced, primary school education, currently using MMT, a heroin user).

Additionally, participants frequently said that a lack of insurance is a major barrier to entering clinics or MMT programs. Having no insurance or being unable to pay for insurance may not be an issue in most developed countries that have a community health system that provides coverage for treating people with addictions, but it is a major problem for patients in Iran.

One patient said, *“I am a daytime construction worker. I only earn money on the days that I find work. Sometimes, I really do not have the money to pay for my methadone. I wish that the insurance would cover my addictin treatment.*” (P14, 46 years old, married, secondary school education, a heroin user).

### Theme #2: Lack of awareness about methadone treatment

Patients generally do not know the nature of MMT and have unrealistic expectations of treatment. The majority of patients think they will be able to put their drug use aside forever and that they will experience just a few months of methadone addiction problems. However, using methadone for only a few months is incompatible with the goals of MMT harm-reduction programs, in which the goal is to increase patients’ health and to avoid physical side effects. Most of the participants felt that they had received inadequate information about methadone. One patient said,

*“Unfortunately I was addicted to heroin for eight months. Now I’m here at the MMT center. I have never received information on MMT at any time. Before I was an addict, I haven’t been in conditions where I’ve been required to connect with addicted people; consequently, I have never been in a condition where I’ve had to hear about methadone.”* (P5; 43 years old, divorced, college education, an opium user).

The majority of the patients also reported a lack of knowledge about the duration and doses of methadone treatment.

*“When I entered the MMT, I thought that I could gradually reduce the dose of methadone and gradually cut off methadone, so that I would quit drugs forever. But after the four years that I have been treated with methadone, I’m still taking methadone. And recently, I found that it might be forever that I will use methadone.”* (P19; 52 years old, married, high school education, a heroin user).

Another participant stated,

*“The doctors never told me that I should use methadone for a long period. They always give us delusive hope... If I had known from the beginning that MMT was prolonged, perhaps I would have never used it.”* (P22; 45 years old, married, primary school education, a crack-heroin user).

### Theme #3 negative attitudes regarding using methadone

Participants had different and mixed beliefs about methadone. Some of these beliefs were negative. A mistrust of MMT and fear of becoming dependent on methadone were observed between participants. Some participants mentioned that they had no desire to be treated with methadone due to the rate of relapse in MMT compared to treatments for other drugs. Of course, this comparison may serve as a justification in the case of patients who do not want to continue treatment, or it could mean that some successful participants in MMT may not be an illustrative or actual sample of patients who have not entered MMT as compared to patients who have entered MMT and also remained in treatment. This viewpoint becomes clearer in the following statement:

*“I have no desire to be treated by methadone. I believe that methadone is more addictive than opioids. One of my friends was on MMT, but he had to use heroin at the same time due to a lack of effect. Therefore, I believe it isn’t effective.”* (P6; 29 years old, single, college education, a crack-heroin user).

Some participants had worries and fears about MMT and believed it was stupid to exchange one drug with another. During MMT, the dose of methadone is reduced at the discretion of the clinic. Some patients have difficulties accepting this because they still feel the need to use drugs. As such, patients experience problems withdrawing from methadone and believe methadone withdrawal is more difficult than heroin withdrawal.

*“Withdrawing from methadone is more difficult than withdrawing from heroin. It causes severe bone pain and emotional pain. It is like an insect biting your body. I don’t know how to explain this experience. Further, one side effect is that you need to sleep more.”* (P12; 40 years old, primary school education, married, crack-heroin user).

As another participant stated,

*“I think that MMT is more addictive than other opioids, and it is more difficult to quit from it. I am not going to use methadone once more due to its addictive nature. I prefer to use other opioids.”* (P8; 34 years old, primary school education, single, heroin user).

### Theme #4 worries about methadone side effects

While the side effects of methadone that were reported by patients who get treatment did not appear to be very strong, they nevertheless caused worries, and worries about the side effects were one of the main reasons for giving up MMT. The level of worry about methadone side effects varied among the participants enrolled in MMT. The most frequently mentioned methadone side effects were sleep disturbances, dizziness, decreased sexual desire, itching, vomiting, bloating, liver disorders, diarrhea, and constipation. Some patients reported that these effects disrupted their family and work life. One interviewee complained,

*“It almost decreased my sexual desire! It was more severe than consuming hashish. Most of the people who have been treated by methadone report the same symptoms as I have. My wife thought that I had a girlfriend. She didn’t understand me. She thought that I was betraying her at that time.”* P10; 41 years old, married, high school education, a crack-heroin user).

In addition, most of the patients had not been informed about the side effects of methadone. Some of them were unable to get information about the side effects from the methadone clinic, due to a lack of professional doctors. However, some of them were also unable to find suitable treatment for methadone side effects in other medical clinics. Some clinics said to the patients that the key solution to these problems was reducing the methadone dose. However, reducing the dosage of methadone might result in mental or physical problems. With regard to these side effects, some patients acknowledged that they received a response to their questions from the methadone clinic staff. One participant stated,

*“At the beginning of the treatment, I did not receive any information about methadone side effects. During seven months using methadone, I faced many problems, including erection problems. I went to the doctor. He said that this was because of the methadone. Unfortunately, I did not get any information beforehand from the clinical staff about the side effects of methadone. I was really afraid about the side effects of it!”* (P14, 46 years old, married, secondary school education, a heroin user).

Others mentioned perspectives on the side effects of methadone that might serve as a warning about future methadone treatment. The following sentences show some of the patients’ viewpoints:

*“Methadone is worse than crack-heroin. It is actually a risky drug. It makes my bones breakable. And as a habit it’s worse than heroin. I would rather take hashish than methadone. I got a friend that was treated with methadone: he used 100 milligrams methadone, and he died after eight months.”* (P1; 55 years old, married, high school education, a heroin user).

### Theme #5: Social stigma ascribed to methadone therapy

Most patients generally reported social stigma toward methadone users as one of the reasons why opioid users did not want to be treated by methadone. The fear of stigma was worsened by society’s negative attitude toward people with addictions. The participants explained their experiences of being rejected once their opioid use became recognized by other persons in society. Additionally, these participants explained that they have low expectations of creating a new, nonstigmatized identity, even after a long period of opioid withdrawal. Patients described that being known as an opioid addict would probably result in a rejection by society. A current opioid user reported the following reason why he rejects using methadone:

*“When my family sees that I use methadone syrup, I am ashamed. Even when they remind me that I must use methadone and I must not forget the syrup, I am embarrassed. Although my family knows that I am treated with methadone, I feel bad.”* (P10; 41 years old, married, high school education, a crack user).

*“Frankly speaking, when I get methadone from the clinics, I am worried that if one of my family members sees me in the MMT center, they will reject me... I’m addicted and I understand that my treatment gives a very bad reputation to my family.”* (P12; 40 years old, primary school education, married, crack user).

Other participants gave the following statements:

*“As a methadone user, the community rejects you. They’re still looking at you as an opioid user. Nobody (except my addicted friends) wants a relationship with me. I’ve become like a wasteful person. My identity is very shameful.”* (P8; 34 years old, primary school education, single, heroin user).

*“There is one more thing about methadone that I want to say: some people look at us in a different manner. Unfortunately, people only look negatively at methadone... People who are not using methadone believe that addicted persons who are getting methadone are still addicted.”* (P12; 40 years old, primary education, married, crack user).

Some participants mentioned that they did not want to be treated by methadone in an MMT or public clinic because it would cause them to be known in public as an addicted person. Three participants explained their viewpoint in the following way:

*“When you go to a methadone clinic, your name is registered. I want my identity to be unknown, so I won’t be able to go to a methadone clinic.”* (P9; 41 years old, single, a heroin user).

“*It’s true that for months I have not used heroin, but I do not feel clean at all. Maybe I’m no longer addicted to heroin, but now I’m a methadone addict. And I know that if people know that I’m addicted to methadone, they do not think so well about me.”* (P13; 41 years old, married, high school education, a crack user).

*“Once at the airport, at an inspection, one of the agents took notice of my methadone pills and reminded my colleagues to carefully check my suitcase. It was as if she was facing a suspect who gave her a bad feeling.”* (P9; 41 years old, single, a heroin user).

### Theme #6 systemic barriers in methadone treatment

Almost all of the patients said there were systemic barriers to treatment with methadone, including lack of support from specialist services, inappropriate behavior with patients, the necessity of regular visits to get methadone, the time-consuming and long duration of the treatment, and strict laws.

Participants stated that they had a tendency to reuse drugs when they faced some behavior from therapists.

“*Every morning, I must go to the methadone center for my treatment and take my daily methadone. I have to do that during my working hours. My boss is not happy with this. I wish they could give some methadone for a week to some patients*.” (P11; 32 years old, single, college education, a heroin user).

“*I do think that therapists have a main role to play, since one might assume that they have experience about addicted persons, but many of them have not behaved well toward us. They could take care of us in a better way, especially in the beginning of treatment with methadone. But they are not taking enough time to provide us with information to treat us. In my opinion, the majority of therapists and physicians have looked at us in a humiliating way... they look from a top-down perspective.”* (P13; 41 years old, married, high school education, a crack user).

Having supportive services, like psychological counseling from therapists, seems to be an essential need for several patients. However, it looks like these services are very inadequate in the MMT clinics. The therapists have a high workload and because they are too busy, they do not offer enough information. Most participants reported that they left MMT clinics without getting essential counseling from a therapist. Some patients complained about the lack of private areas for individual consultation with therapists. As one participant said,

“You know, I did not get any education or counseling. I hoped that the physicians would talk with me for a moment, but they didn’t. In my opinion, having more counseling would be extremely valued. I was hoping that the therapists could explain how to avoid drugs... Now I feel like I just get the dose of methadone every day without any patient’s rights.” (P8; 34 years old, primary school education, single, heroin user).

*“My family asked me whether I went today to a center to get methadone. ... I said yes... I went to a gas station though… [laughing] I think the clinics are like petrol stations. We take methadone so that we can stand and continue our life. No adviser.... No teacher.... No warning.”* (P9; 41 years old, single, a heroin user).

Patients also complained about the length of the treatment, the necessity of regular visits to get methadone, and the time-consuming aspects of the treatment process, which are all interlinked:

*“The main problem is that methadone treatment takes a while. We also have to be present a long time beforehand to get methadone at the MMT. If the duration of treatment would be shorter, I think that we would cope better with the difficulties of the treatment process.”* (P16; 33 years old, college education, single, crack user).

Logistical barriers and rigid rules presented another kind of systemic barrier that was reported by participants. Some patients reported that a short time after the beginning of treatment with methadone, they returned to using their previous drug of choice because the open hours of the MMT clinics did not match their working hours. Furthermore, employed patients had many problems when going out of the city for business. Due to these problems, many of the participants explained that they had no intention to enter a long-term MMT program. Two participants explained their viewpoint in the following way:

*“I am a normal worker. My working hours are from 7 am to 5 pm. I need this job to continue my life. On the other hand, the clinicians work from 8 am to 5 pm. Because I must go to work at 7 am, I cannot come to the clinic. I have to choose either to go to work or to go to a clinic for treatment. I think it is my biggest problem.”* (P7; 58 years old, divorced, high school education, currently using MMT, a heroin user).

Another reason for patients’ dissatisfaction regarding treatment with methadone was problems related to the quality of methadone syrup.

*“The first time that I received methadone syrup, I had lost my craving and consumption. I was happy to consume it, and I did not have any symptoms from taking it. But after a while, it was as though the methadone quality was changed... waterier… As if it did not correspond with the previous dose. When I told this to the technician, he said that the pharmaceutical company had been changed. I had really severe constipation. My mouth was dry, and this was so unbearable that I had to stop taking methadone anymore.”* (P9; 41 years old, single, a heroin user).

*“I have no problem with methadone tablets, but I don’t like syrup. Generally, I have problems with eating, but methadone tablets are easier to transport. It is easy to measure the doses. For example, it is obvious how much 5 mg is... but for me, 5 ml syrup is difficult to measure. I was afraid to drink too much or too little. But most Iranian MMT centers offer patients syrup and not tablets.”* (P22; 45 years old, married, primary school education, a crack user).

## Discussion

To the best of our knowledge, this is the first study that qualitatively presents the obstacles to attendance and adherence to MMT programs as perceived by Iranian opioid-dependent patients. Based on the personal stories of the drug-dependent patients in MMT programs, we identified six themes contributing to barriers to retention in MMT.

### Financial barriers related to methadone treatment

The majority of the interviewees reported the cost *of treatment* as being a major obstacle to attendance and continuing at MMT programs, especially for unemployed and low-income patients. These findings are in line with other studies [13, 19, 28] that reported financial problems as a key factor that dissatisfies patients with treatments for drug use. Despite the large number of Iranian drug-dependent patients seeking treatment, insurance schemes do not cover all drug treatments in Iran. It seems that *reducing or* eliminating drug-treatment costs would encourage more drug-dependent patients to attend drug treatment and to remain longer in the programs, which could improve treatment outcomes [30, 31]. Since MMT as a harm-reduction strategy benefits both patients and society enormously [32], it would be highly effective to allocate more financial resources to Iranian drug-treatment programs like MMT. Insurance coverage should be improved, and it might be beneficial to provide financial support to patients, such as a public transportation card for free or at a discount rate.

### Lack of awareness about methadone treatment

It is clearly shown that there are misconceptions regarding MMT [5]. A large number of interviewees had some unrealistic expectations of the drug treatment. Not accepting opioid dependence as a chronic and relapsing disorder, they expected methadone therapy to be a curative treatment that would last only for a short time and treat their drug disorder quickly and completely. Lack of awareness about MMT has been reported several times [33, 34]. Also, negative attitudes toward MMT have been reported in some studies [13, 35]. MMT as a prolonged replacement treatment rather than a therapeutic treatment is a barrier to attending such a program and complying with its agenda [36]. As described by Xu, people with drug dependence prefer short-term treatments [14]. A previous study demonstrated that health education interventions may reduce patients’ misconceptions about MMT, but can also raise the probability of dropout from treatment [5]. However, Csete and colleagues [37] suggested that decision-making about treatment agendas should be carried out only by treatment practitioners, and it seems that receiving *thorough,* clear information regarding the nature of treatment is within the patient’s rights*.* In this study, some of the interviewees reported that practitioners or physicians demonstrated inappropriate, disruptive, and unfriendly behaviors, which discouraged them from continuing treatment. Since effective patient-clinician relationships improve outcomes and adherence to therapy [38], one strategy would be to provide *communication* skills *training* programs for Iranian practitioners.

### Negative attitudes regarding methadone use

Our findings revealed that some of the interviewees were skeptical about the effectiveness of MMT. In fact, few of the interviewees believed that MMT could be a helpful strategy for drug treatment. As suggested by Babrora and colleagues, drug-dependent patients have less trust in treatment systems [19]. One possible explanation could be the high relapse rate among MMT participants. A study conducted between 2007 and 2011 in Iran found high rates of relapse among opioid-dependent patients participating in MMT, with 64% of patients relapsing within six months after treatment admission [39]. As suggested by previous studies, the effectiveness of MMT has been increased by comprehensive services such as psychological consultation, motivational enhancement therapy, behavioral intervention, and structured relapse prevention [16, 40, 41].

Ward has stressed the important role of staff in making MMT programs efficacious [42]. Thus, a key factor regarding the effectiveness of Iranian MMT programs could be using well-trained staff who have good communication skills and detailed knowledge about patients’ concerns about MMT therapy. An effective and efficient way of offering training in physician-patient communication is in the form of seminars or workshops where strategies are covered for improved communication in a relatively short period of time. Also, we found that the viewpoints of the patients’ family members and relatives toward MMT could play a leading role in patients’ motivation to comply with an MMT program. In fact, social support not only improves therapeutic responses but also has an effect on retention in treatment. Higher social support is related to higher retention and completion of treatment [43, 44]. Also, social support plays a role in decreasing stigmatization. Having no or too little support from family or from MMT workers may increase the risk of the *recurrence* of *addiction* following a period of remission [45]. The process of treatment is very stressful, and it is hard to stay clean without support.

Several interviewees reported that their family members were concerned about the long duration of MMT. One explanation might be that long-term treatment could make family members rather tired of supporting dependent patients financially and emotionally. It seems that many of the patients’ families are not familiar with the fact that methadone treatment is a prolonged replacement therapy and not a short-term curative therapy. Given *that academics have suggested* that family support is related to retention for MMT [46], it could be strongly suggested that all family members be educated about MMT programs. Family members’ training is possible by holding group meetings and family counseling sessions whenever patients are referred to methadone treatment. Educational interventions for family members could change their attitudes toward MMT programs and increase their knowledge about them, which could in turn result in more support from family members to follow MMT therapy.

### Worries about methadone side effects

Most of the interviewees indicated that the side effects of methadone were obstacles that hindered attending an MMT program. Several interviewees had experienced methadone therapy to have harmful effects on their liver performances, teeth, and sexual performance. Also, some complained of constipation during the treatment period. This finding is consistent with a recent study of Stancliffin that pointed out the negative effects of methadone on teeth and bones [47, 48]. A recent study conducted in the US found that drug-dependent patients believed that methadone therapy might have adverse health effects [4]. Also, some interviewees undergoing MMT programs were worried about methadone hangover symptoms and a danger of becoming methadone dependent. This result has been confirmed in other studies [4, 18, 33, 47, 49, 50].

Not only do *these concerns encourage* a preference for other treatment styles [14], but they also increase dropout rates among MMT participants. Some interviewees participating in Iranian MMT programs expressed their preference for not complying with the treatment agenda. In fact, some of them used to reduce the methadone doses gradually because they misunderstood the adverse effects of high doses of methadone. In contrast, some dependent patients participating in MMT programs increased their methadone dose without a medical prescription in order to eliminate the withdrawal symptoms of retention. Therefore, we suggest providing patients with effective modern (e.g., acupuncture) and traditional medical treatments (e.g., painkillers in the short period after starting treatment) to reduce the side effects of MMT.

### Social stigma ascribed to methadone therapy

The results of our study further indicated that the stigma on MMT remains a great barrier to attendance at MMT programs. Some of the interviewees in this study dropped out of treatment because of suffering from severe embarrassment when going to MMT centers. These findings are in line with those of other studies [16]. Some studies have revealed that methadone-related stigma experienced by drug users could influence their treatment decision-making [51, 52]. Thus, policy makers should be responsible for providing a noncritical social environment for drug treatment systems in order to remove or reduce stigma among the public community toward Iranian individuals seeking these treatment services. This could be done through mass media or social media interventions.

### Systemic barriers in methadone treatment

Iran has made remarkable progress in the establishment of MMT clinical centers in recent years. It is estimated that at least 1600 MMT clinics deliver treatment services for more than 159,000 opioid-dependent patients in Iran [1]. As a result, Iran is one of the most successful countries in implementing MMT. There are several systemic barriers in the country that hinder attendance of MMT programs.

Appearing to receive a daily dose in the first months of treatment is a main perceived barrier to attending MMT given the prolonged length of treatment. As mentioned by some interviewees, daily referral to MMT clinics causes patients serious difficulties in different areas related to their jobs and families. Furthermore, daily referral is a waste of patients’ time and makes them worry about being seen. Our findings suggest that treatment services should consider options such as take-home doses, which may improve treatment compliance by eliminating the need for daily attendance. Also, as shown by Gao and colleagues, take-home dose services increase retention time and improve treatment outcomes [53]. Furthermore, some of the patients’ complaints against treatment systems are related to the quality of methadone syrup provided by different pharmaceutical companies. In addition, some drug-dependent patients preferred to use methadone tablets rather than syrup due to the ease of carrying and using them, whereas methadone is distributed in the form of syrup in all MMT clinics in Iran.

### Limitation

One limitation of the present study is that the small sample size limits our ability to generalize the results to other drug users. As such, the findings of the study should be interpreted with caution.

## Conclusions

This study aimed to explore barriers to and misconceptions about MMT in Iranians with drug dependence. Our findings suggest that Iranian people with addictions and their families should be better informed about the duration of MMT programs and the side effects of MMT. In addition, addicts might be helped by reducing the costs of MMT.

## Abbreviations

BMT: Buprenorphine maintenance therapy
HIV: Human immunodeficiency virus
MMT: Methadone maintenance therapy
OTS: Opioid treatment services
UNODC: United Nations Office on Drugs and Crime
